# Utility of heparin-binding protein following cardiothoracic surgery using cardiopulmonary bypass

**DOI:** 10.1038/s41598-023-48457-y

**Published:** 2023-12-07

**Authors:** Emilia Johannesson, Clara Erixon, Niklas Sterner, Louise Thelaus, Alain Dardashti, Johan Nilsson, Sigurdur Ragnarsson, Adam Linder, Igor Zindovic

**Affiliations:** 1 Department of Cardiothoracic Surgery, Clinical Sciences, Lund University, Skane University Hospital, 221 85 Lund, Sweden; 2 Division of Infection Medicine, Department of Clinical Sciences Lund, Faculty of Medicine, Lund University, Lund, Sweden; 3 Thoracic Surgery and Bioinformatic Research Unit, Department of Translational Medicine, Lund University, Lund, Sweden; 4 Department of Thoracic and Vascular Surgery, Skảne University Hospital, Lund, Sweden

**Keywords:** Diagnostic markers, Predictive markers, Prognostic markers, Bacterial infection

## Abstract

Cardiothoracic surgery using cardiopulmonary bypass (CPB) triggers an inflammatory state that may be difficult to differentiate from infection. Heparin-binding protein (HBP) is a candidate biomarker for sepsis. As data indicates that HBP normalizes rapidly after cardiothoracic surgery, it may be a suitable early marker of postoperative infection. We therefore aimed to investigate which variables influence postoperative HBP levels and whether elevated HBP concentration is associated with poor surgical outcome. This exploratory, prospective, observational study enrolled 1475 patients undergoing cardiothoracic surgery using CPB, where HBP was measured at ICU arrival. Patients with HBP in the highest tercile were compared to remaining patients. Multivariable logistic regressions were performed to identify factors predictive of elevated HBP and 30-day mortality. Overall median HBP was 30.0 ng/mL. Patients undergoing isolated CABG or surgery with CPB-duration ≤ 60 min had a median HBP of 24.9 ng/mL and 23.2 ng/mL, respectively. Independent predictors of elevated postoperative HBP included increased EuroSCORE, prolonged CPB-duration and high intraoperative temperature. Increased HBP was an independent predictor of 30-day mortality. This study confirms the promising characteristics of HBP as a biomarker for identification of postoperative sepsis, especially after routine procedures. Further studies are required to investigate whether HBP may detect postoperative infections.

## Introduction

Cardiothoracic surgery with the use of cardiopulmonary bypass (CPB) triggers an acute phase reaction and causes a systemic inflammatory response syndrome (SIRS), potentially resulting in organ dysfunction^1–3^. The inflammatory response caused by the CPB is related to the exposure of blood to the non-physiological surface of the CPB-circuit and is further amplified by surgical trauma itself, endotoxemia, and ischemia–reperfusion injury^1–3^. This inflammatory response is associated with an increased risk of postoperative complications, including infections, myocardial and neurological dysfunction, bleeding disorders, renal and respiratory failure, and multiple organ failure^1–4^.

Up to 22% of patients undergoing cardiac surgery will suffer from postoperative nosocomial infections, increasing the risk of prolonged hospitalization and mortality^4^. In clinical practice, it is difficult to differentiate between postoperative, surgically induced inflammation and postoperative infection, both clinically and by means of laboratory analyses. Therefore, a reliable tool for early identification of postoperative infections is necessary.

Heparin-binding protein (HBP) is a neutrophil-derived protein, which mainly resides within azurophilic granules. It promotes inflammation by acting as a chemoattractant, as an activator of monocytes, and by inducing increased capillary permeability via interaction with the endothelium^5^. HBP is an indicator of neutrophil activation and causes vascular leakage, which is strongly intertwined with the pathophysiology of severe infections. Therefore, HBP has gained considerable attention over the last decades, primarily as a potential biomarker for sepsis^5,6^. Previous research has shown HBP with a threshold of 30 ng/mL to be a robust biomarker for sepsis with an ability to predict development of organ dysfunction with high prognostic accuracy, even before clinical signs are apparent^6–8^.

In cardiothoracic surgery, HBP concentration has been shown to increase substantially following the use of CPB but normalizes rapidly after CPB cessation^9,10^. Hence, HBP may have the potential to identify infections at an early postoperative stage. The aim of the present study was to assess which pre- or intraoperative variables were associated with increased postoperative HBP levels at intensive care unit (ICU)-arrival and thus to identify those patients who would be suitable for early postoperative screening for infections using HBP. In addition, we sought to investigate whether postoperative HBP measured directly at ICU-arrival could be used to predict postoperative outcomes and mortality after cardiothoracic surgery.

## Results

### Study population

**Figure 1:**
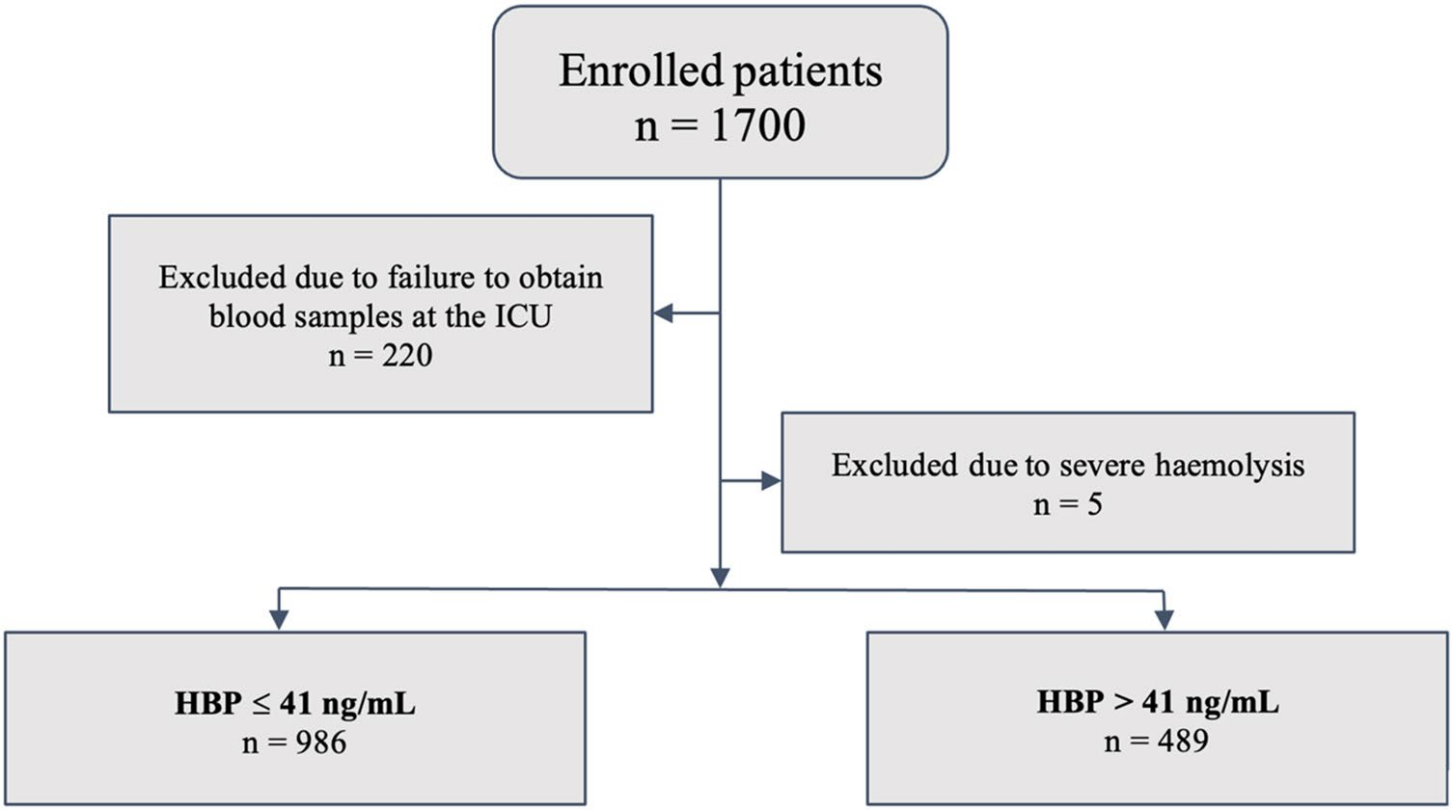
A flowchart describing the study design, excluded patients along the way, and distribution amongst the sub-groups.

A total of 1700 consecutive patients were enrolled in the study between 1st of February 2020 and 22nd of September 2021, 220 of whom were excluded due to failure to obtain blood samples at the ICU, and additional five of whom were excluded due to severe sample haemolysis, making the total number of participants in the study 1475 (Fig. 1). The tercile of the study population with the highest measured HBP concentrations rounded up to the next integer (> 41 ng/mL) comprised 489 patients, and the remaining two-thirds consisted of 986 patients.

### Patient characteristics

The median age was 68 years (range: 18–90), and 25% of the patients were female. Almost half of the patients (n = 730) underwent isolated CABG, 668 of whom were patients with a non-critical preoperative state who underwent non-acute surgery. The median CPB duration was 74 (55–106) minutes, and the median aortic cross-clamping time was 48 (34–70) minutes. A total of 495 patients underwent surgery with CPB-duration ≤ 60 min.

### Baseline patient data

Preoperative characteristics of the study population are displayed in Table 1. Patients with an HBP > 41 ng/mL more often presented with PAP > 60 mmHg (8% *vs* 3*%*; *p* < 0.001) compared to the low HBP-group. They were also more likely to have had previous cardiac surgery (15% *vs* 6%; *p* < 0.001), ongoing endocarditis (7% *vs* 3%; *p* < 0.001), acute surgery (15% *vs* 5%; *p* < 0.001), and were more likely to be in a critical preoperative state (8% *vs* 3%; *p* < 0.001) when compared to the low HBP-group. Patients with HBP > 41 ng/mL had a higher logistic EuroSCORE (6.5 [3.5–17.3] *vs* 3.9 [2.1–7.7];* p* < 0.001) and higher preoperative creatinine concentration (86 [73–107] μmol/L *vs* 81 [71–93] μmol/L;* p* < 0.001) compared to the low HBP-group. Patients with an HBP > 41 ng/mL were less likely to have a history of smoking (36% *vs* 42%; *p* = 0.028), hypertension (54% *vs* 63%; *p* = 0.001) or diabetes mellitus (21% *vs* 27%; *p* = 0.019) compared to the low HBP-group. All the p-values in this paragraph were generated using the Chi-square test, unless regarding logistic EuroSCORE and preoperative creatinine concentrations which were generated using the Mann–Whitney U test.

**Table 1:** Preoperative characteristics of the study population.

Variable	All (n = 1475)	HBP > 41 (n = 489)	HBP ≤ 41 (n = 986)	p	Missing
Age	68 (60–75)	69 (59–76)	68 (60–74)	0.170*	0
Female sex	362 (24.5)	131 (26.8)	231 (23.4)	0.158	0
BMI	27.40 (± 4.63)	27.17 (± 4.81)	27.51 (± 4.54)	0.187°	4
History of smoking	503 (40.1)	146 (35.7)	357 (42.2)	0.028	220
Hypertension	879 (59.8)	263 (53.9)	616 (62.7)	0.001	5
Previous cardiac surgery	132 (8.9)	72 (14.7)	60 (6.1)	< 0.001	0
Previous stroke	102 (6.9)	36 (7.4)	66 (6.7)	0.634	0
Diabetes mellitus	369 (25.0)	104 (21.3)	265 (26.9)	0.019	0
COPD	127 (8.6)	49 (10.0)	78 (7.9)	0.174	0
Peripheral vascular disease	83 (5.6)	25 (5.1)	58 (5.9)	0.546	0
Left ventricular ejection fraction (%)				< 0.001	
> 50	1005 (68.1)	300 (61.3)	705 (71.5)		0
30–50	341 (23.1)	133 (27.2)	208 (21.1)		
< 30	129 (8.7)	56 (11.5)	73 (7.4)		
NYHA class				< 0.001	
I	416 (28.2)	109 (22.3)	307 (31.1)		0
II	599 (40.6)	183 (37.4)	416 (42.2)		
III	350 (23.7)	134 (27.4)	216 (21.9)		
IV	110 (7.5)	63 (12.9)	47 (4.8)		
PAP > 60 mmHg	64 (4.3)	39 (8.0)	25 (2.5)	< 0.001	0
Preoperative creatinine concentration (µmol/L)	83 (72–97)	86 (73–107)	81 (71–93)	< 0.001*	0
Endocarditis	63 (4.3)	35 (7.2)	28 (2.8)	< 0.001	0
Acute surgery	124 (8.4)	72 (14.7)	52 (5.3)	< 0.001	0
Preoperative critical state	70 (4.7)	41 (8.4)	29 (2.9)	< 0.001	0

NOTE. Values are expressed as numbers (%), mean (± standard deviation) or median (interquartile range).

HBP, heparin binding protein; BMI, body mass index; COPD, chronic obstructive pulmonary disease; NYHA, New York Heart Association; PAP, pulmonary artery pressure.

Statistical tests: Chi-Square test, Mann Whitney U test (*) and Student´s t test (°).

### Intraoperative data

Intraoperative characteristics of the study population are depicted in Table 2. The duration of CPB (97 [68–139] min *vs* 66 [50–89] min; *p* < 0.001) and cross-clamping (61 [41–87] min *vs* 44 [32–60] min;* p* < 0.001) was significantly longer among patients with an HBP > 41 ng/mL. Intraoperative temperature nadir was lower (35.7 [34.0–36.0] °C *vs* 36.0 [35.0–36.0] °C; *p* < 0.001) among patients with an HBP > 41 ng/mL. Median HBP concentration for patients undergoing surgery with a CPB duration ≤ 60 min (n = 495) was significantly lower compared to those with a CPB duration > 60 min (n = 980) (23.2 [17.3–34.7] ng/mL *vs* 35.8 [23.3–52.9] ng/mL; *p* < 0.001. Of patients with CPB time ≤ 60 min, 77.8% had HBP levels < 30 ng/mL. All the p-values in this paragraph are generated using the Mann–Whitney U test, unless regarding the variable CPB duration > 60 min which was generated using the Chi-square test.

**Table 2:** Intraoperative characteristics of the study population.

Variable	All (n = 1475)	HBP > 41 (n = 489)	HBP ≤ 41 (n = 986)	p	Missing
Main surgical procedure					
Isolated CABG	730 (49.5)	152 (31.1)	578 (58.6)	< 0.001	0
Isolated AVR	233 (15.8)	82 (16.8)	151 (15.3)		
CABG + AVR	97 (6.6)	41 (8.4)	56 (5.7)		
Mitral valve repair	90 (6.1)	39 (8.0)	51 (5.2)		
Mitral valve replacement	41 (2.8)	20 (4.1)	21 (2.1)		
Aortic surgery	61 (4.1)	26 (5.3)	35 (3.5)		
Aortic surgery + AVR	79 (5.4)	36 (7.4)	43 (4.4)		
Double valve procedure	46 (3.1)	29 (5.9)	17 (1.7)		
Heart transplant	31 (2.1)	27 (5.5)	4 (0.4)		
Lung transplant	10 (0.7)	10 (2.0)	0 (0)		
Other procedure	57 (3.9)	27 (5.5)	30 (3.0)		
Duration of CPB (min)	74 (55–106)	97 (68–138.5)	66 (50–89)	< 0.001*	0
Aortic cross-clamping time (minutes)	48 (34–70)	61 (41–87)	44 (32–60)	< 0.001*	0
Temperature nadir (°C)	36.0 (35.0–36.0)	35.7 (34.0–36.0)	36.0 (35.0–36.0)	< 0.001*	9
Procedure with circulatory arrest	62 (4.2)	32 (6.5)	30 (3.0)	0.002	0

NOTE. Values are expressed as numbers (%) or median (interquartile range).

HBP, heparin binding protein, CABG, coronary artery bypass graft; AVR, aortic valve replacement; CPB, cardiopulmonary bypass.

Statistical tests: Chi-Square test and Mann Whitney U test (*).

**Figure 2:**
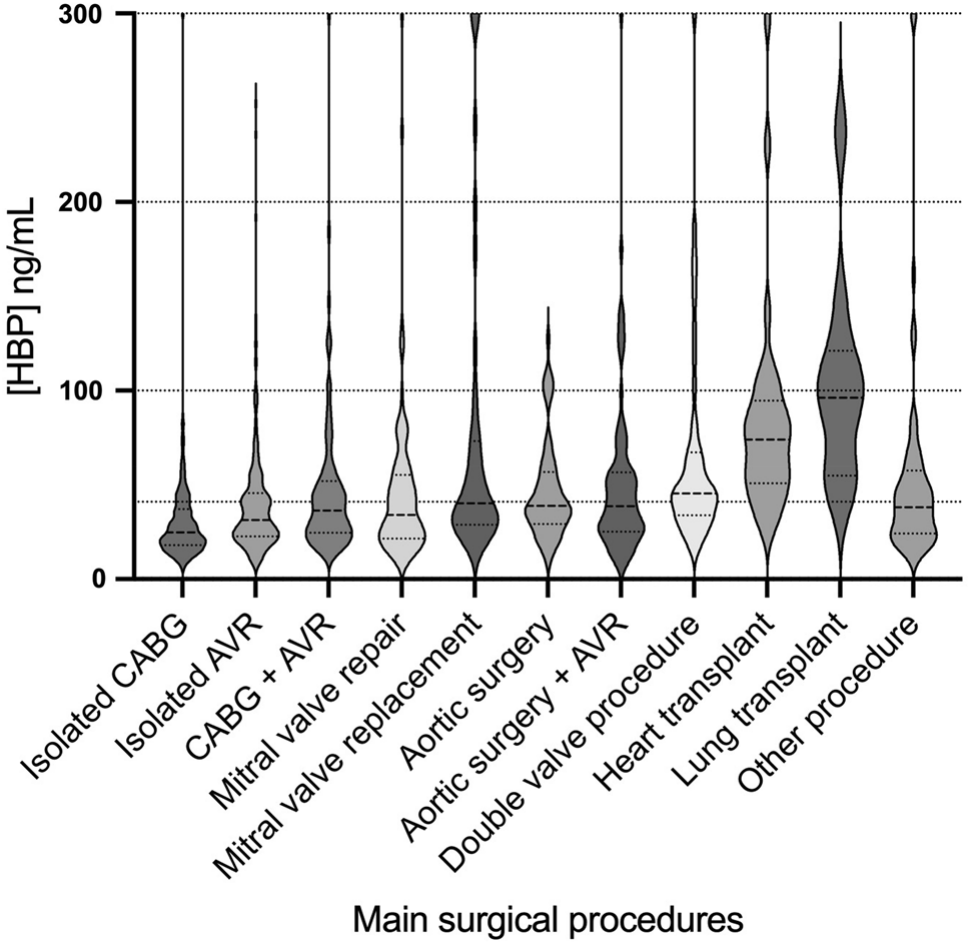
Violin plots for HBP concentration according to surgical procedure performed. NOTE. The width of each violin corresponds with the approximate frequency of data points in each region. The three solid lines within the violins represent the 25th, 50th, and 75th percentiles. The lower respectively the upper adjacent value are presented as the most central thinnest parts of the violins. The bold, dotted reference line on the y-axis represents HBP = 30 ng/mL. The laboratory analysis apparatus measured HBP concentrations within the range of 5.90–300.00 ng/mL). HBP, heparin binding protein; CABG, coronary artery bypass graft; AVR, aortic valve replacement.

The distribution and numbers of surgical procedures performed and HBP concentrations across these different surgical procedures are displayed in Table 2 and Fig. 2. At ICU-arrival, the overall median HBP concentration was 30.0 (20.6–46.6) ng/mL. Patients undergoing isolated CABG (n = 730) had a median HBP level of 24.9 (18.0–37.2) ng/mL, and 63.6% had HBP levels < 30 ng/mL. Patients who were not in a preoperative critical state undergoing elective isolated CABG (n = 668) had a median HBP level of 24.5 (17.7–36.5) ng/mL.

### Postoperative patient data, complications, and mortality

Postoperative outcomes of the study population are presented in Table 3. Patients with HBP > 41 ng/mL more often suffered from postoperative complications, including postoperative stroke (4% *vs* 2%; p = 0.002), postoperative kidney injury (15% *vs* 3%; p < 0.001), postoperative infections (8% *vs* 3%; p < 0.001), multiple organ failure (3% *vs* 0%; p < 0.001), and new-onset atrial fibrillation (29% *vs* 25%; p = 0.044). Patients with HBP > 41 ng/mL had significantly higher 30-day mortality compared to remaining patients (3.5% *vs* 0.5%; *p* < 0.001). All the p-values in this paragraph are generated using the Chi-square test.

**Table 3:** Postoperative characteristics of the study population.

Variable	All (n = 1475)	HBP > 41 (n = 489)	HBP ≤ 41 (n = 986)	p	Missing
Peak CKMB concentration (µg/L)	18 (12–29)	24 (15–46)	16 (12–23)	< 0.001*	9
Peak lactate concentration (mmol/L)	2.20 (1.70–3.00)	2.50 (2.00–3.50)	2.10 (1.60–2.70)	< 0.001*	7
Peak CRP concentration (mg/L)	121 (57–193)	140 (83–211)	102 (48–183)	< 0.001*	975
Peak WBC count (× 10^9^/L)	12.7 (10.5–15.2)	13.7 (11.1–17.4)	12.3 (10.3–14.6)	< 0.001*	200
Peak creatinine concentration (µmol/L)	98 (81–129)	107 (88–169)	94 (80–119)	< 0.001*	7
Total dose (μg) norepinephrine administered per kilogram	12.47 (2.63–48.40)	30.43 (5.09–145.07)	8.33 (1.94–30.65)	< 0.001*	0
Duration of norepinephrine administration (h)	15 (9–20)	17 (12–33)	14 (8–18)	< 0.001*	0
Total dose (mg) dobutamine administered per kilogram	0 (0–0.79)	0 (0–2.36)	0 (0–0.19)	< 0.001*	0
Duration of dobutamine administration (h)	0 (0–10)	0 (0–16)	0 (0–4)	< 0.001*	0
Time on ventilator (h)	4.33 (2.98–7.10)	5.43 (3.43–14.04)	4.05 (2.82–6.02)	< 0.001*	0
> 48 h on ventilator	64 (4.3)	46 (9.4)	18 (1.8)	< 0.001	0
Postoperative infection	65 (4.4)	39 (8.0)	26 (2.6)	< 0.001	0
Postoperative stroke	35 (2.4)	20 (4.1)	15 (1.5)	0.002	0
Postoperative CRRT or dialysis	48 (3.3)	35 (7.2)	13 (1.3)	< 0.001	0
Postoperative kidney injury	103 (7.0)	72 (14.7)	31 (3.1)	< 0.001	0
Postoperative multiple organ failure	19 (1.3)	16 (3.3)	3 (0.3)	< 0.001	0
Reoperation due to bleeding	50 (3.4)	21 (4.3)	29 (2.9)	0.176	0
Postoperative IABP	45 (3.1)	24 (4.9)	21 (2.1)	0.003	0
New-onset atrial fibrillation	386 (26.2)	144 (29.4)	242 (24.5)	0.044	0
30-day mortality	22 (1.5)	17 (3.5)	5 (0.5)	< 0.001	0

NOTE. Values are expressed as numbers (%) or median (interquartile range).

HBP, heparin binding protein; CKMB, creatine kinase myocardial band; CRP, C-reactive protein; WBC, white blood cell; CRRT, continuous renal replacement therapy; IABP, intra-aortic balloon pump.

Statistical tests: Chi-Square test and Mann Whitney U test (*).

The time on ventilator (5.4 [3.4–14.0] h *vs* 4.1 [2.8–6.0] h; *p* < 0.001) was significantly longer for those patients with an HBP > 41 ng/mL. Furthermore, the group with HBP > 41 ng/mL received a higher total dose of norepinephrine (30.4 [5.1–145.1] μg/kg *vs* 8.3 [1.9–30.7] μg/kg; *p* < 0.001) and required longer duration of norepinephrine treatment (17 [12–33] h *vs* 14 [8–18] h; *p* < 0.001). All the p-values in this paragraph are generated using the Mann–Whitney U test.

The peak concentration of all the other inflammatory markers measured at the ICU was significantly higher in patients with an HBP > 41 ng/mL: lactate (2.5 [2.0–3.5] mmol/L *vs 2*.1 [1.6–2.7] mmol/L; *p* < 0.001), C-reactive protein (CRP) (140 [83–211] mg/L *vs* 102 [48–183] mg/L; *p* < 0.001), and white blood cell (WBC) count (13.7 [11.1–17.4] × 10^9^/L *vs* 12.3 [10.3–14.6] × 10^9^/L; *p* < 0.001). This also applied to creatine kinase myocardial band (CKMB) (24 [14–46] μg/mL *vs* 16 [12–23] μg/mL; *p* < 0.001) and creatinine (107 [88–169] μg/mL *vs* 93.5 [80–119] μmol/L; *p* < 0.001). All the p-values in this paragraph are generated using the Mann–Whitney U test. Results from correlation analyses between HBP plasma concentration and postoperative variables are presented in Supplementary Table 1. These did not demonstrate any moderate or strong correlations between HBP concentration and assessed variables.

### Univariable- and multivariable analyses

**Figure 3:**
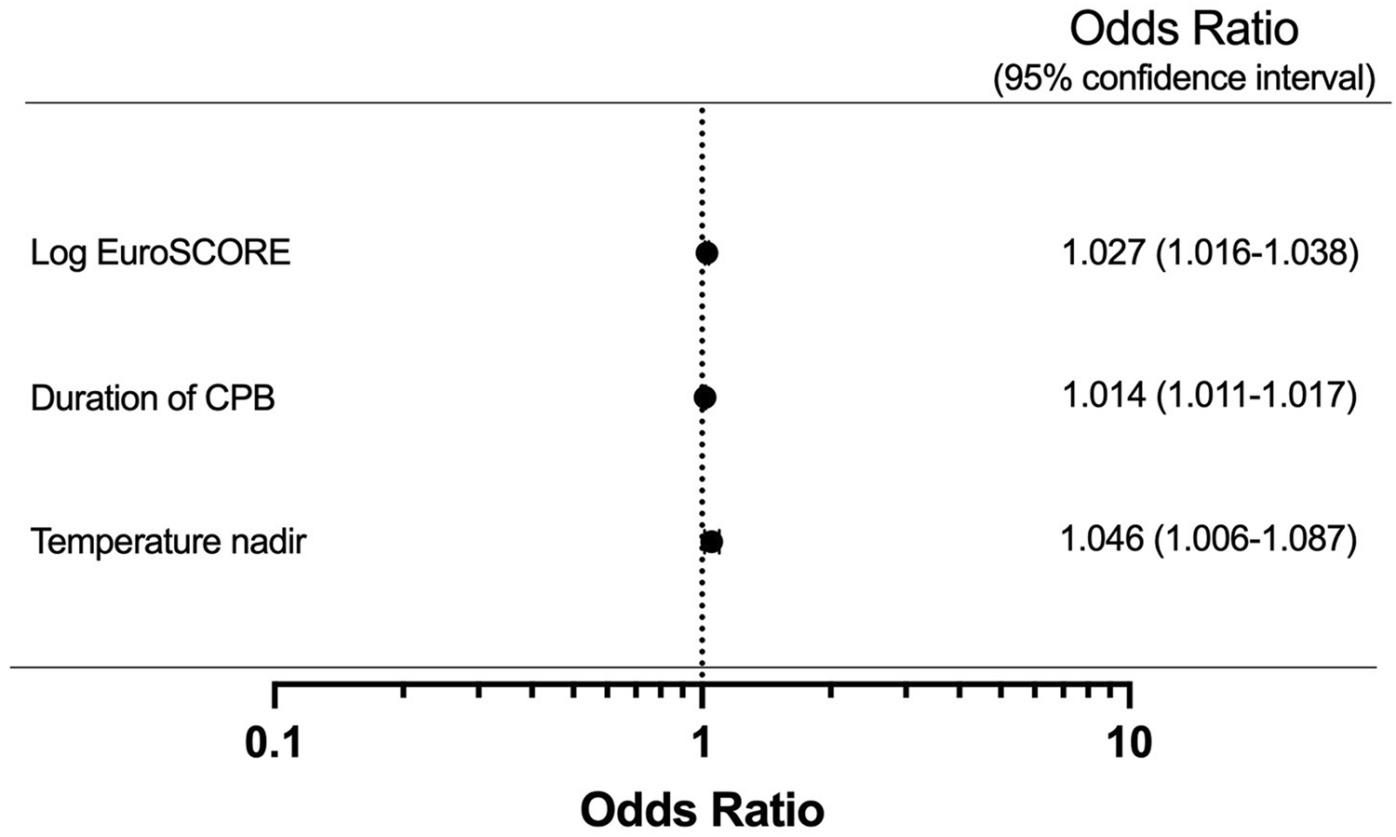
Forests plots representing multivariable logistic regression for independent predictors of an HBP concentration > 41 ng/mL. NOTE. Values are expressed odds ratios (OR) (central dot) and 95% confidence intervals (95% CI) (whiskers). Variables presented in the figure are only the ones significant in the multivariable analysis model. For the continuous variables, the presented OR’s are associated with one-unit increment for each given variable**:** Log EuroSCORE (%), duration of CPB (minutes) and temperature nadir (°C). This figure presents results derived from the core model. EuroSCORE, European system for cardiac operative risk evaluation; CPB, cardiopulmonary bypass. Statistical tests: multivariable logistic regression.

**Figure 4:**
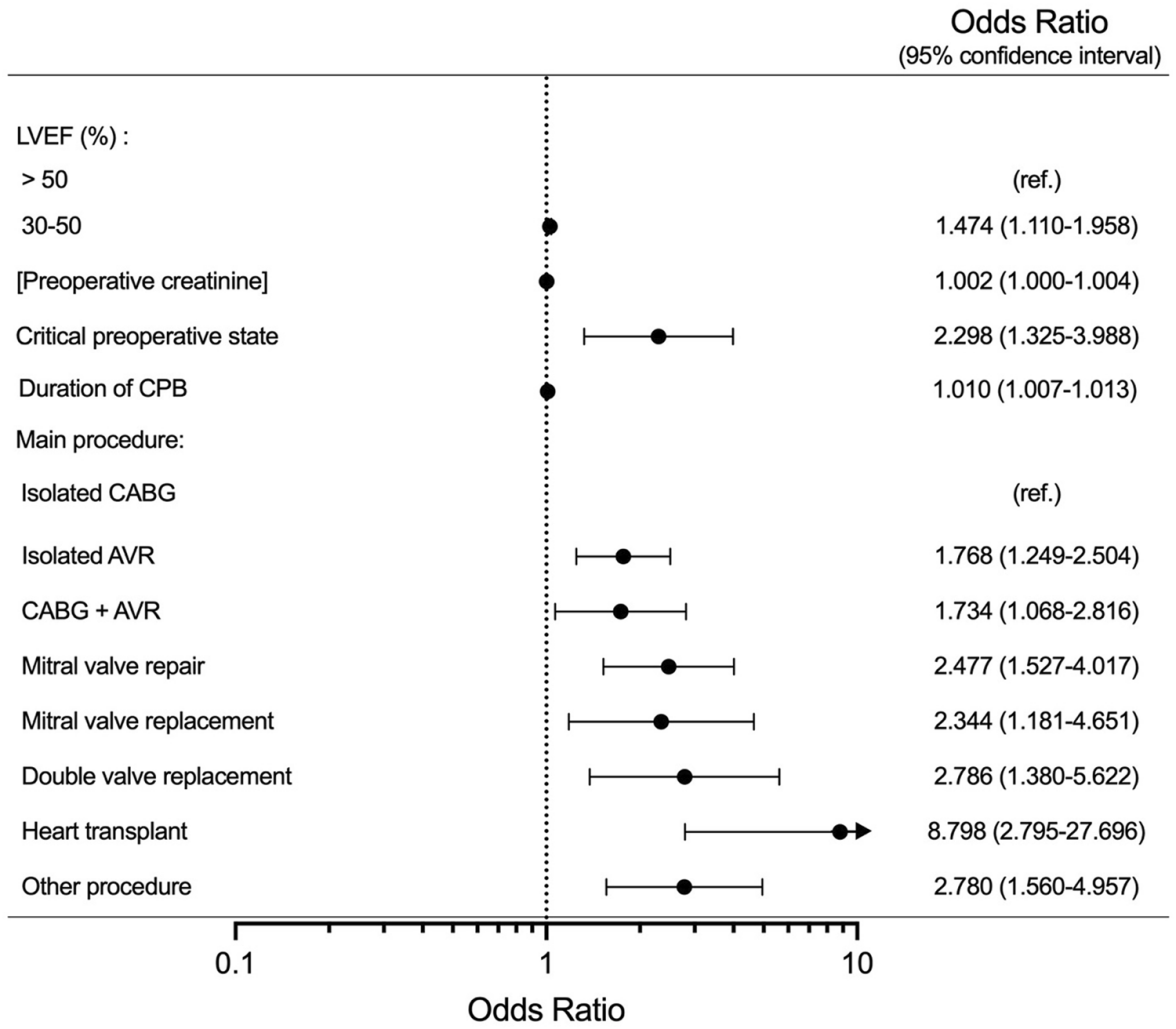
Forests plots representing multivariable logistic regression for independent predictors of an HBP concentration > 41 ng/mL. NOTE. Values are expressed odds ratios (OR) (central dot) and 95% confidence intervals (95% CI) (whiskers). Variables presented in the figure are only the ones significant in the multivariable analysis model. For the continuous variables, the presented OR’s are associated with one-unit increment for each given variable**:** preoperative creatinine (µmol/L) and duration of CPB (minutes). LVEF, left ventricular ejection fraction; CPB, cardiopulmonary bypass; CABG, coronary artery bypass graft; AVR, aortic valve replacement. Statistical tests: multivariable logistic regression.

The correlation coefficient between duration of CPB and aortic cross-clamping time was strong (R = 0.816, R2 = 0.666), and therefore the latter one was excluded from future analyses. The independent predictors of elevated HBP concentration are presented in Figs. 3 and 4. Our core model identified logistic EuroSCORE (OR 1.027, CI 1.016–1.038; *p* < 0.001), duration of CPB (OR 1.014, CI 1.011–1.017; *p* < 0.001) and increasing intraoperative temperature nadir (OR 1.046, CI 1.006–1.087; *p* = 0.009) as independent predictors of elevated HBP concentration. In the model where logistic EuroSCORE was excluded and its included variables analysed separately, preoperative creatinine concentration (OR 1.002, CI 1.000–1.004; *p* = 0.017), LVEF 30–50% (OR 1.474, CI 1.110–1.958;* p* = 0.007), critical preoperative state (OR 2.298, CI 1.325–3.988; *p* = 0.003) and several surgical procedures (isolated AVR (OR 1.768, CI 1.249–2.504; p = 0.001), CABG + AVR (OR 1.734, CI 1.068–2.816; *p* = 0.026), mitral valve repair (OR 2.477, CI 1.527–4.017; p < 0.001), mitral valve replacement (OR 2.344, CI 1.181–4.651; *p* = 0.015), double valve procedure (OR 2.786, CI 1.380–5.622; p = 0.004) and heart transplant (OR 8.798, CI 2.795–27.696; *p* < 0.001)) were also identified to be predictive of HBP > 41 ng/mL. In this model, duration of CPB (OR 1.010, CI 1.007–1.013; *p* < 0.001) was once again identified as an independent predictor of elevated HBP concentration.

**Figure 5:**
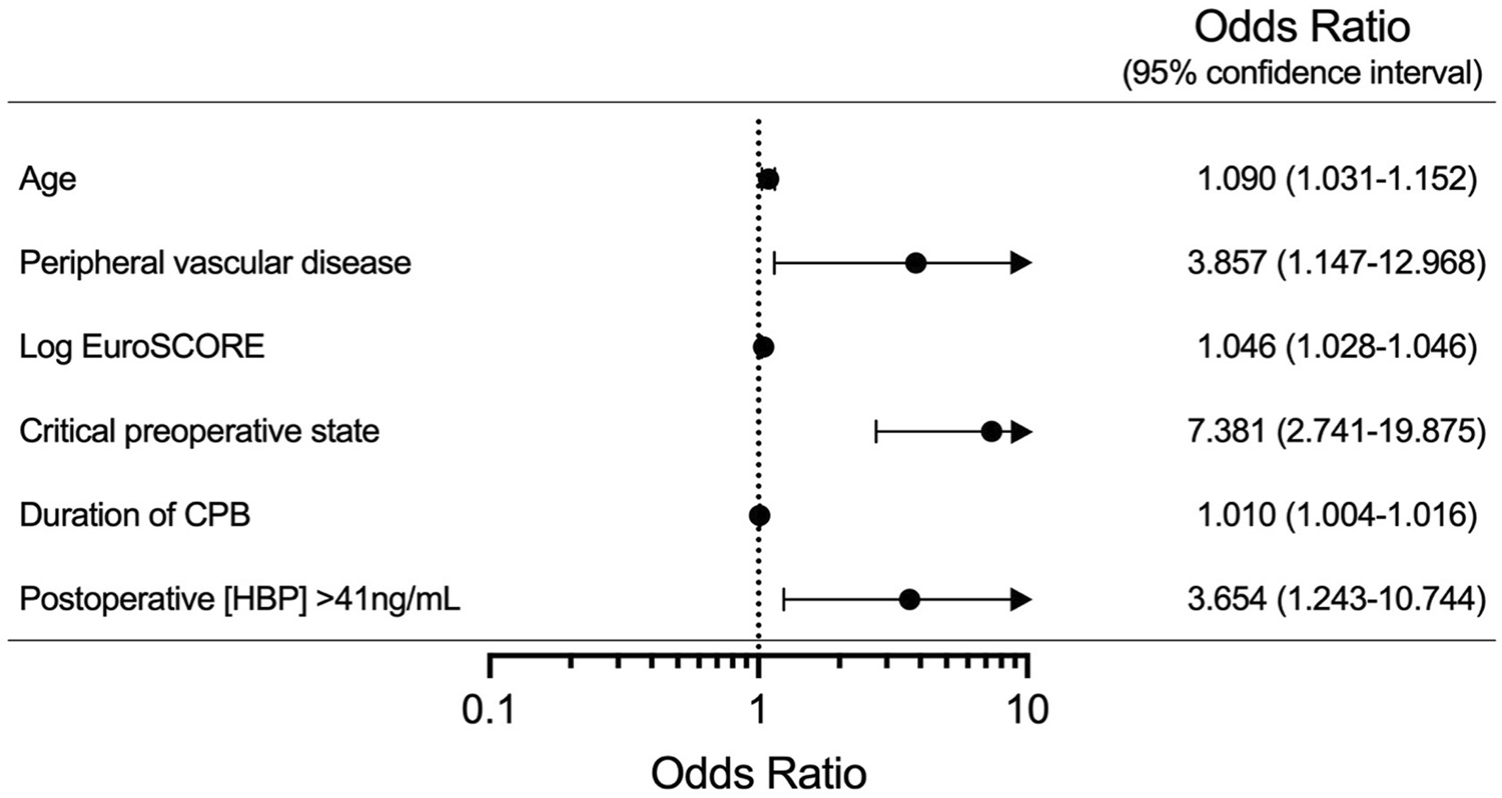
Forests plots representing multivariable logistic regression for independent predictors of 30-day mortality. NOTE. Values are expressed odds ratios (OR) (central dot) and 95% confidence intervals (95% CI) (whiskers). Variables presented in the figure are only the ones significant in the multivariable analysis model. For the continuous variables, the presented OR’s are associated with one-unit increment for each given variable: age (years), Log EuroSCORE (%), duration of CPB (minutes) and HBP concentration (ng/mL). PAP, pulmonary artery pressure; EuroSCORE, European system for cardiac operative risk evaluation; CPB, cardiopulmonary bypass; HBP, heparin-binding protein. Statistical tests: multivariable logistic regression.

Furthermore, HBP > 41 ng/mL (OR 3.654, CI 1.243–10.744; *p* = 0.019) and HBP concentration per ng/mL increment (OR 1.009, CI 1.003–1.014; *p* = 0.001) were identified as independent predictors of 30-day mortality (Fig. 5 and Supplementary Table 2). All the p-values in this paragraph are generated using multivariable logistic regression.

### Sensitivity analysis

We observed mild-moderate haemolysis in 127 samples (in addition to the five samples with severe haemolysis). Although, median HBP concentration in the group with mild-moderate haemolysis was 45.6 (30.4–72.8) ng/mL compared to 29.1 (20.2–44.9) ng/mL (*p* < 0.001, Mann–Whitney U-test) in the group with no visual haemolysis, our analyses showed no significant changes in our results related to mild-moderate haemolysis. Therefore, the only samples excluded from further analyses were those with severe haemolysis, in accordance with the guidelines of the manufacturer (Supplementary Tables 3–5).

## Discussion

To the best of our knowledge, this is the largest report to investigate HBP levels in patients undergoing cardiothoracic surgery^9–11^. In this study, we showed that the group of patients with the highest HBP had a seven-fold increase in 30-day mortality compared to remaining patients. We also identified several factors that influenced postoperative HBP concentration, most of which were linked to complex surgical procedures. Furthermore, we demonstrated that approximately half of patients undergoing surgery using cardiopulmonary bypass had HBP levels lower than the previously defined threshold for sepsis detection (30 ng/mL), upon ICU-arrival^7^. In routine procedures (*e.g.* isolated, elective CABG and procedures with CPB duration ≤ 60 min) the proportion of patients with HBP concentration < 30 ng/mL at ICU-arrival was higher.

The effects of CPB use and sepsis share pathophysiological mechanisms in terms of proinflammatory cytokine release, neutrophil activation, hyperpermeability, and subsequently, tissue damage. Since HBP is released as a reaction to infection and progression from infections to sepsis, it is plausible to assume that HBP dynamics would be affected by the SIRS caused by the use of CPB^1,2,5,12,13^. In this study, CPB duration was an independent predictor of elevated HBP levels at ICU-arrival. However, the correlation between HBP concentration and CPB duration was weak.

The fact that postoperative HBP concentration is affected by CPB is to its detriment in terms of HBP’s ability to predict postoperative infections among patients undergoing cardiothoracic surgery. However, this is not different from other markers of inflammation such as procalcitonin (PCT) and CRP, which also are elevated after the use of CPB. Aouifi et al.^14^ demonstrated that peak PCT concentration occurred on the first postoperative day and reached baseline at postoperative day five. CRP peaked 2–3 days after surgery and remained elevated until day eight, and Fardin et al.^15^ showed an increasing trend in WBC during the first 48 h after cardiac surgery with the use of CPB. Taken together, these biomarkers are unreliable when measured directly after the end of surgery. In contrast, HBP reaches its peak concentration during cardiopulmonary bypass and is more rapidly cleared from the blood^9^. In the majority of cases, HBP reaches levels below the cutoff for sepsis at ICU arrival and returns to baseline levels on the first postoperative day^9^. This indicates that the effect of surgery-induced inflammation subsides rapidly enough to allow HBP to be used as a reliable marker of early postoperative infection, especially if measured serially^16^. However, since sepsis is a heterogenous and complex disease, no sole biomarker is likely to adequately detect the potentially septic patient. More likely, a variety of biomarkers will be required to accurately identify those patients who need more careful monitoring^17^. This is supported by Linder et al.^7^ who demonstrated that a combination of CRP, PCT, WBC, lactate, and HBP had superior predictive ability to detect sepsis than any one biomarker used alone.

CPB duration has several confounders, the most important being surgical complexity. Surgical complexity, in turn, is determined both by the surgical procedure performed and the preoperative clinical state of the patient. Here, we demonstrated that several preoperative variables predicted elevated postoperative HBP levels: increased creatinine levels, critical state, high logistic EuroSCORE, and reduced LVEF. These variables have been described as confounding factors for elevated HBP in earlier studies but with other study populations^11^.

An overall view of the data available indicates that factors contributing to a deteriorated general condition affect postoperative HBP levels. Although Sterner et al.^18^ showed that preoperative HBP levels were similar between patients undergoing CABG and complex procedures, patients in a preoperative critical state may be under a state of neutrophil activation, contributing to higher postoperative HBP concentrations. However, this is speculative as we did not measure preoperative HBP concentration.

In addition, our results showed that HBP levels increased with the technical complexity of the procedure. The highest HBP concentrations were observed in patients undergoing double valve procedures and heart- or lung transplants, whereas the lowest HBP concentrations were observed in patients undergoing isolated CABG and surgical procedures with CPB duration ≤ 60 min. In these groups, a majority had an HBP concentration below 30 ng/mL, a previously described cutoff for the detection of sepsis^7^. This implies that HBP may be used as a biomarker for detection of sepsis at an early postoperative stage in a large proportion of patients undergoing routine cardiothoracic surgery.

The main focus of HBP in previous research has been its potential as a predictor of complications in general and infections in particular. HBP concentrations are elevated in sepsis and correlate with the development of hypotension and circulatory failure^5^. Furthermore, HBP is able to predict septic organ dysfunction up to three days before clinical signs become apparent^7^. Although this was an exploratory study aiming to identify factors that may affect the specificity of HBP in an early postoperative stage, our results showed that patients in the highest HBP tercile more frequently suffered postoperative infections, stroke, atrial fibrillation, and multiple organ failure. Moreover, 30-day mortality was seven times higher in the highest HBP tercile compared to the remaining patients*.* In addition, elevated HBP concentration was identified as an independent predictor of increased 30-day mortality, corresponding well with previous findings^6,19^. Although this is in line with previous research, this study was not designed to assess these endpoints in greater detail. Therefore, we lack valuable information about these complications, one example being the timing of the complication in relation to the collected blood sample. Therefore, future studies designed to assess these endpoints specifically are required, preferably by means of serial HBP measurements.

Vasodilatory shock, or vasoplegic syndrome, following CPB may affect as many as half of all patients undergoing major cardiac surgery, and its pathophysiology is similar to that of sepsis^20^. To maintain adequate tissue perfusion, these patients exhibit increased postoperative vasopressor requirement, and norepinephrine has long been considered the first line of therapy^20^. With this in mind, our observation that patients in the group with the highest HBP levels (> 41 ng/mL) required more than three times the total dose of norepinephrine compared to patients in the remaining two-thirds of the study population is in accordance with previous findings. These earlier findings demonstrated that the overall HBP levels during the first 3 days of ICU-stay was associated with the total norepinephrine requirement^8^. However, the duration of norepinephrine administration only differed by 3 h on average. This may be explained by the rapid clearance of HBP, and that a few hours may be all that is required for the vasodilatation in the group with HBP > 41 ng/mL to subside. Our findings are in line with previous observations that elevated HBP concentration is correlated with fluid overload and hypervolemia and consequently associated with increased permeability and vascular leakage^18^.

This study is limited by HBP concentration only being investigated at one specific time point. Several confounders may influence the interpretation of a single value, for example the natural dynamics of HBP, differences in baseline characteristics of the study population, and differences in logistics surrounding surgery and blood sample collection. Furthermore, our results indicate a large degree of interindividual variability in terms of HBP levels and therefore, serial measurement of biomarkers would have been preferable as it has been proven to increase prognostic value^8^.

As mentioned previously, 30 ng/mL has been suggested as a cutoff value for sepsis prediction^7^. However, other studies have proposed different threshold values^6,7,21^. Conceivably, this reflects a difference between studied cohorts, but it could also represent differences in lab techniques and sample handling. In this study, we used a relatively new method for HBP measurement. This method has been shown to correlate well with HBP measurements using the ELISA technique^22^ but, to the best of our knowledge, no studies have determined cutoff values for detection of sepsis using the Joinstar analyser. However, when validating the precision performance of the Joinstar-device, it consistently generated results in the upper part of the reference interval, yet with excellent linearity. Therefore, if our results were to differ from previous studies, it is likely that the concentrations generated in our analyses are higher than they would have been using the ELISA method.

HBP released following neutrophil lysis, because of sample haemolysis, is a possible source of error in the measured HBP concentration. In this study, we used Hb > 4.0 mg/mL as the threshold for exclusion in accordance with the manufacturer’s instructions. However, other studies have decided on lower cutoff values for significant degree of haemolysis^8^. We did, however, perform a sensitivity analysis that showed no major interference on results.

Despite these limitations, this study is the largest to investigate HBP concentrations after any surgical procedure and relies on a well-defined study population with high completeness of data and study follow-up.

In conclusion, postoperative concentrations of HBP are affected by several factors, most of which are related to the complexity of the surgical procedure performed. However, approximately half of the patients undergoing cardiothoracic surgery with cardiopulmonary bypass have HBP concentrations upon arrival to the ICU below the previously suggested cutoff for identifying sepsis. We could also demonstrate that the lowest HBP values were observed in patients undergoing isolated CABG or procedures with an CPB duration ≤ 60 min, suggesting that HBP may be especially useful as an early biomarker of postoperative infections in an early postoperative stage in patients undergoing routine cardiac surgery. Finally, we could show that patients with the highest HBP levels were at significantly higher risk of postoperative complications and that HBP was an independent predictor of 30-day mortality.

## Methods

### Study design and setting

This was a prospective, observational, exploratory, single centre study. The study population consisted of patients undergoing cardiothoracic surgery with the use of cardiopulmonary bypass at the Department of Cardiothoracic Surgery, Skane University Hospital, Lund, Sweden. Patient enrolment occurred between 1st of February 2020 and 22nd of September 2021. Patients were followed until 30 days after surgery. Patients aged 18 years or older were eligible for enrolment in the study. The sole exclusion criterion was if the surgical procedure was performed without the use of CPB. Individual informed patient consent was obtained from all the study participants and the study was approved by the Swedish Ethical Review authority (ref. 2019-00210, 5th of March 2019). The research was performed in accordance with relevant guidelines and regulations. The Swedish Ethical Review authority and the Institutional Data Monitoring committee at Skane University Hospital did approve the experimental protocols.

### Definitions

Critical preoperative state was defined using the European system for cardiac operative risk evaluation (EuroSCORE)^23^. EuroSCORE was calculated by the responsible surgeon. Acute surgery was defined as surgery carried out within 24 h from decision to perform surgery. Preoperative hypertension was defined as blood pressure > 140/90 mmHg or the use of anti-hypertensive drugs.

Postoperative infection was confirmed by clinical or radiological assessment or by positive cultures from the suspected site of infection. Multiple postoperative organ failure was defined as failure of two or more organ systems. Postoperative kidney injury was defined as postoperative creatinine concentration more than twice as high as the preoperatively measured value in accordance with the RIFLE (risk, injury, failure, loss of kidney function and end-stage kidney disease) criteria for kidney injury^24^. Postoperative cardiac failure was defined as inotropes administered > 24 h postoperatively and/or postoperative requirement of an intra-aortic balloon pump (IABP). Respiratory failure was defined as ventilator requirement > 48 h. Postoperative atrial fibrillation was considered confirmed if observed during the postoperative hospital stay.

### Surgical procedures

The surgical procedures performed were categorized as follows: coronary artery bypass grafting (CABG), aortic valve replacement (AVR), CABG + AVR, mitral valve repair, mitral valve replacement, aortic surgery, aortic surgery + AVR, double valve procedure (surgery to at least two heart valves), heart transplant, and lung transplant. “Other procedures” were procedures of varying surgical complexity not included in remaining surgical categories (*e.g.* atrial septal defect (ASD) closure, post infarction ventricular septal defect (VSD) repair, and pulmonary valve conduit replacement).

The anaesthetic and surgical protocols at our department have been described previously^9^. All patients were heparinized to an activated clotting time (ACT) > 480 s before CPB initiation. To measure perioperative ACT the Hemochron Jr. Signature + (ITC, Edison, NJ, US) was used. To calculate perioperative heparin levels as well as the amount of protamine needed to neutralize the heparin levels before CPB termination, the HepProCalc software was used. The CPB system consisted of non-coated polyvinyl chloride and silicone tubing, a Stöckert S5 roller pump (LivaNova, London, United Kingdom), a hard-shell open venous reservoir, and an FX25 Terumo oxygenator (Terumo, Tokyo, Japan).

### Sample collection

Venous blood samples for determination of HBP were collected in ethylenediaminetetraacetic (EDTA) vacuum blood collection tubes from a central venous catheter upon postoperative ICU-arrival. The blood samples were centrifuged at 2200 × g for 10 min after which the samples were separated and stored in aliquots at -80 °C until analysed. The plasma samples were thawed to room temperature and thoroughly mixed before use.

### Laboratory analysis

HBP concentrations in plasma were measured using a Heparin Binding Protein Detection Kit (Fluorescence Dry Quantitative Immunoassay, Jet-iStar 800, Joinstar, Zhejiang, China), which is based on fluorescence immunoassay technology, for rapid detection of HBP. Mellhammar et al.^22^ validated this method by measuring HBP using both the Joinstar point of care assay and enzyme-linked immunoassay (ELISA) technique. Mellhammar and colleagues found a satisfactory correlation between the methods (with an R-value of 0.83). In accordance with the manufacturer’s recommendations, each sample was visually screened for severe haemolysis (haemoglobin (Hb) > 4.0 mg/mL) or turbidity, which resulted in the exclusion of five samples. Before analysing the remaining blood samples, a validation of the HBP Joinstar point of care assay was performed. Accuracy, lower limit detection, linearity, and precision were verified to be within the ranges specified by the manufacturer (the apparatus measured HBP-concentrations within the range of 5.9–300.0 ng/mL). Mellhammar et al.^22^ previously described these methods in detail. Furthermore, lower limit detection and linearity were measured and evaluated after each block of 300 samples to assure that the performance of our measurements was consistent.

Remaining laboratory tests were analysed according to clinical routine at the Department of Laboratory Medicine, Skane University Hospital, Lund, Sweden. CKMB was routinely collected on the day of surgery and postoperative day one. CRP and WBC were collected on postoperative days one, two and four. Additional analyses were performed clinically indicated.

### Statistical analysis

Before statistical analysis, the study population was divided into two sub-groups: one group comprised patients with the highest tercile of HBP concentration (rounded up to the next integer, which meant a cutoff at 41 ng/mL), and a second group consisted of the remaining two-thirds of patients.

Continuous, non-parametric variables are presented as medians and interquartile ranges (IQR) and evaluated using the Mann–Whitney U test. Continuous, parametric variables are presented as means and standard deviations (± standard deviation (SD)) and evaluated using the Student’s t-test. Categorical variables are presented as numbers of observations (n) and percentages (%). Proportions were juxtaposed and compared using the Chi-square test unless the number of observations was equal to, or less than 5, in which case the Fisher’s exact test was used. Two-sided-*p-*values less than 0.05 were considered statistically significant.

Correlation analyses between the plasma concentration of HBP and postoperative variables were performed using the Pearson correlation yielding a correlation coefficient (R). Additionally, a correlation analysis between duration of CPB and aortic cross-clamping time was performed in the same way.

Predictors of elevated concentration of HBP (HBP > 41 ng/mL) were assessed using univariable logistic regression analyses for all baseline and intraoperative characteristics, except for smoking (excluded due to numerous missing values). Subsequently, a backward selection multivariable logistic regression relying on complete case analysis was performed using all factors with *p* < 0.1 in the univariable test. We conducted two multivariable logistic regressions. One including EuroSCORE and the other with the separate components of the score. The model including the EuroSCORE was the core model for the remaining analyses. The results were presented using OR’s with 95% confidence intervals (CI) for each variable. The same method was used to identify independent predictors of 30-day mortality, and separate models were used to generate the OR’s for HBP concentration as a continuous variable and the categorical variable “HBP > 41 ng/mL”, where the latter constituted the core model.

To assess whether mild or moderate degrees of haemolysis affected HBP levels measured with the Joinstar-kit, a sensitivity analysis was performed after all samples with visual haemolysis were excluded.

Statistical analyses were performed using the SPSS 27 software (IBM Corp. Released 2020. IBM SPSS Statistics for Macintosh, Version 27.0. Armonk, NY: IBM Corp).

### Supplementary Information

Supplementary Tables.

## Data Availability

The datasets generated and analysed during the current study are not publicly available due to limitations in the ethical approval but are available from the corresponding author on reasonable request.

## Abbreviations

CPB: Cardiopulmonary bypass
SIRS: Systemic inflammatory response syndrome
HBP: Heparin-binding protein
ICU: Intensive care unit
EuroSCORE: European system for cardiac operative risk evaluation
IABP: Intra-aortic balloon pump
CABG: Coronary artery bypass graft
AVR: Aortic valve replacement
ACT: Activated clotting time
ELISA: Enzyme-linked immunoassay
R: Correlation coefficient
Hb: Haemoglobin
IQR: Interquartile range
N: Number of observations
LVEF: Left ventricular ejection fraction
PAP: Pulmonary artery pressure
OR: Odds ratio
CI: Confidence interval
CRP: C-reactive protein
WBC: White blood cell
NO: Nitric oxide
PCT: Procalcitonin
