# Supplementary material for: Utility of heparin-binding protein following cardiothoracic surgery using cardiopulmonary bypass
Source: Sci Rep. 2023 Dec 7;13:21566. doi: 10.1038/s41598-023-48457-y (PMC10700527; doi:10.1038/s41598-023-48457-y)
Supplement: Supplementary file 1 — Supplementary Tables. [file 41598_2023_48457_MOESM1_ESM.doc]

# Supplementary material

**Supplementary Table 1. *Pearson correlation test between HBP concentration and selected postoperative variables***

| **Variable** | **Pearson correlation coefficient (R)** | ***p*** | **n** |
| --- | --- | --- | --- |
|  |  |  |  |
| Duration of CPB (minutes) | 0.356 | < 0.001 | 1475 |
| Time on ventilator (hours) | 0.232 | < 0.001 | 1475 |
| Total dose of norepinephrine  administered (μg) per kilogram | 0.304 | < 0.001 | 1475 |
| Total dose of dobutamine administered (mg) per kilogram | 0.216 | < 0.001 | 1475 |
| Duration of norepinephrine administration (hours) | 0.287 | < 0.001 | 1475 |
| Duration of dobutamine administration (hours) | 0.234 | < 0.001 | 1475 |

| Postoperative maximum lactate concentration (mmol/L) | 0.353 | < 0.001 | 1468 |
| --- | --- | --- | --- |
| Postoperative maximum CRP concentration (mg/L) | 0.283 | < 0.001 | 500 |
| Postoperative maximum WBC count (x10^9^/L) | 0.260 | < 0.001 | 1275 |

***NOTE.*** *Pearson correlation tests between postoperative HBP concentrations and postoperative variables.****Abbreviations:*** *CPB, cardiopulmonary bypass; CRP, C-reactive protein; WBC, white blood cell.*

***Statistical tests:*** *Pearson correlation test.*

**Supplementary Table 2. *Univariable and multivariable logistic regression to identify independent predictors of 30-day mortality***

| **Variable** | **Univariable analysis**  **(OR (95% CI))** | **P-value** | **Multivariable analysis (OR (95% CI))** | **P-value** |
| --- | --- | --- | --- | --- |
|  |  |  |  |  |
| Age | 1.087 (1.029-1.148) | 0.003 | 1.090 (1.031-1.152) | 0.003 |
| Female sex | 1.443 (0.584-3.568) | 0.427 |  |  |
| BMI | 0.926 (0.838-1.023) | 0.131 |  |  |
| Hypertension | 0.971 (0.412-2.286) | 0.946 |  |  |
| Previous cardiac surgery | 2.300 (0.767-6.900) | 0.137 |  |  |
| Previous stroke | 2.159 (0.628-7.422) | 0.222 |  |  |
| Diabetes mellitus | 1.729 (0.719-4.154) | 0.221 |  |  |
| COPD | 2.403 (0.801-7.212) | 0.118 |  |  |
| Peripheral vascular disease | 3.865 (1.278-11.691) | 0.017 | 3.857 (1.147-12.968) | 0.029 |
| Left ventricular ejection fraction (%): |  |  |  |  |
| >50 | (ref.) | 0.037 | (ref.) |  |
| 30-50 | 2.085 (0.788-5.522) | 0.139 |  |  |
| <30 | 4.012 (1.349-11.929) | 0.012 |  |  |
| PAP>60 | 3.603 (1.038-12.505) | 0.043 |  |  |
| Logistic EuroSCORE (per 1% increment) | 1.051 (1-034-1.069) | < 0.001 | 1.046 (1.028-1.064) | < 0.001 |
| Preoperative creatinine concentration (µmol/L) (per 1 µmol/L increment) | 1.002 (0.998-1.006) | 0.315 |  |  |
| Endocarditis | 5.250 (1.723-16.000) | 0.004 |  |  |
| Acute surgery | 4.243 (1.629-11.047) | 0.003 |  |  |
| Critical preoperative state | 12.820 (5.186-31.696) | < 0.001 | 7.381 (2.741-19.875) | < 0.001 |
| Main procedure: |  |  |  |  |
| Isolated CABG | (ref.) | 0.141 | (ref.) |  |
| Isolated AVR | 0.389 (0.048-3.127) | 0.375 |  |  |
| CABG + AVR | 1.900 (0.398-9.080) | 0.421 |  |  |
| Mitral valve repair | 1.014 (0.125-8.202) | 0.990 |  |  |
| Mitral valve replacement | 4.628 (0.951-22.529) | 0.058 |  |  |
| Aortic surgery | 3.059 (0.635-14.735) | 0.163 |  |  |
| Aortic surgery + AVR | 0 | 0.997 |  |  |
| Double valve procedure | 6.297 (1.613-24.584) | 0.008 |  |  |
| Heart transplant | 6.224 (1.265-30.623) | 0.025 |  |  |
| Lung transplant | 0 | 0.999 |  |  |
| Other procedure | 1.612 (0.198-13.115) | 0.655 |  |  |
| Duration of CPB (per 1 minute increment) | 1.011 (1.007-1.016) | < 0.001 | 1.010 (1.004-1.016) | 0.002 |
| Cross-clamp time (per 1 minute increment) | 1.013 (1.005-1.022) | 0.003 |  |  |
| Temperature nadir (per 1° C increment) | 0.928 (0.855-1.007) | 0.074 |  |  |
| Procedure with circulatory arrest | 2.322 (0.530-10.162) | 0.263 |  |  |
| Postoperative HBP concentration > 41 ng/mL | 7.067 (2.591-19.269) | < 0.001 | 3.654 (1.243-10.744) | 0.019 |
| Postoperative HBP concentration (per 1 ng/mL increment) | 1.011 (1.006-1.016) | < 0.001 | 1.009 (1.003-1.014) | 0.001 |
|  |  |  |  |  |

***NOTE.*** *Values in the table are expressed odds ratios (OR) and 95 % confidence intervals (95% CI).****Abbreviations:*** *BMI, body mass index; COPD, chronic obstructive pulmonary disease;* EuroSCORE, *European system for cardiac operative risk evaluation*; *PAP, pulmonary artery pressure; CABG, coronary artery bypass graft; AVR, aortic valve replacement; CPB, cardiopulmonary bypass.*

***Statistical tests:*** *univariable logistic regression and multivariable logistic regression.*

**Supplementary Table 3.** ***Sensitivity analysis - preoperative characteristics of the study population***

| **Variable** | **All**  (n = 1348) | **HBP > 41** (n = 411) | **HBP** ≤ **41** (n = 937) | ***p*** | **Missing** |
| --- | --- | --- | --- | --- | --- |
|  |  |  |  |  |  |
| Age | 68 (60-75) | 68 (59-75) | 68 (60-74) | 0.605* | 0 |
| Female sex | 324 (24.0) | 106 (25.8) | 218 (23.3) | 0.318 | 0 |
| BMI | 27.33 (±4.62) | 27.13 (±4.82) | 27.41 (±4.53) | 0.307*°* | 4 |
| History of smoking | 458 (39.8) | 117 (33.8) | 341 (42.4) | 0.007 | 197 |
| Hypertension | 805 (59.9) | 218 (53.2) | 587 (62.9) | < 0.001 | 5 |
| Previous cardiac surgery | 116 (8.6) | 62 (15.1) | 54 (5.8) | < 0.001 | 0 |
| Previous stroke | 93 (6.9) | 29 (7.1) | 64 (6.8) | 0.880 | 0 |
| Diabetes mellitus | 343 (25.4) | 88 (21.4) | 255 (27.2) | 0.024 | 0 |
| COPD | 109 (8.1) | 33 (8.0) | 76 (8.1) | 0.960 | 0 |
| Peripheral vascular disease | 79 (5.9) | 22 (5.4) | 57 (6.1) | 0.599 | 0 |
| Left ventricular ejection fraction (%): |  |  |  |  |  |
| > 50 | 908 (67.4) | 245 (59.6) | 663 (70.8) | < 0.001 | 0 |
| 30-50 | 316 (23.4) | 115 (28.0) | 201 (21.5) |  |  |
| < 30 | 124 (9.2) | 51 (12.4) | 73 (7.8) |  |  |
| NYHA class: |  |  |  |  |  |
| I | 383 (28.4) | 89 (21.7) | 294 (31.4) | < 0.001 | 0 |
| II | 554 (41.1) | 157 (38.2) | 397 (42.4) |  |  |
| III | 316 (23.4) | 116 (28.2) | 200 (21.3) |  |  |
| IV | 95 (7.0) | 49 (11.9) | 46 (4.9) |  |  |
| PAP > 60 mmHg | 59 (4.4) | 35 (8.5) | 24 (2.6) | < 0.001 | 0 |
| Logistic EuroSCORE | 4.40 (2.27- 9.46) | 6.35 (3.50-16.81) | 3.75 (2.01-7.64) | < 0.001* | 0 |
| Preoperative creatinine concentration (µmol/L) | 83 (72-97) | 87 (74-109) | 81 (71-93) | < 0.001* | 0 |
| Endocarditis | 57 (4.2) | 30 (7.3) | 27 (2.9) | < 0.001 | 0 |
| Acute surgery | 103 (7.6) | 55 (13.4) | 48 (5.1) | < 0.001 | 0 |
| Preoperative critical state | 58 (4.3) | 32 (7.8) | 26 (2.8) | < 0.001 | 0 |

***NOTE.*** *Prior analysis, 132 samples with haemolysis were excluded. Values are expressed as numbers (%), mean (± standard deviation) or median (interquartile range).****Abbreviations:*** *HBP, heparin binding protein; BMI, body mass index; COPD, chronic obstructive pulmonary disease; NYHA, New York Heart Association; PAP, pulmonary artery pressure; EuroSCORE, European system for cardiac operative risk evaluation.*

***Statistical tests:*** *Chi-Square test, Mann Whitney U test (*) and Student´s t test (°).*

**Supplementary Table 4. *Sensitivity analysis - intraoperative characteristics of the study population***

| **Variable** | | **All**  (n = 1348) | | **HBP > 41** (n = 411) | | **HBP** ≤ **41** (n = 937) | | ***p*** | | **Missing** |
| --- | --- | --- | --- | --- | --- | --- | --- | --- | --- | --- |
|  | |  | |  | |  | |  | |  |
| Main surgical procedure: | |  | |  | |  | |  | |  |
| Isolated CABG | 711 (52.7) | | 137 (33.3) | | 574 (61.3) | | < 0.001 | | 0 | |
| Isolated AVR | 207 (15.4) | | 71 (17.3) | | 136 (14.5) | |  | |  | |
| CABG + AVR | 86 (6.4) | | 34 (8.3) | | 52 (5.5) | |  | |  | |
| Mitral valve repair | 75 (5.6) | | 29 (7.1) | | 46 (4.9) | |  | |  | |
| Mitral valve replacement | 34 (2.5) | | 17 (4.1) | | 17 (1.8) | |  | |  | |
| Aortic surgery | 44 (3.3) | | 17 (4.1) | | 27 (2.9) | |  | |  | |
| Aortic surgery + AVR | 65 (4.8) | | 28 (6.8) | | 37 (3.9) | |  | |  | |
| Double valve procedure | 40 (3.0) | | 24 (5.8) | | 16 (1.7) | |  | |  | |
| Heart transplant | 30 (2.2) | | 26 (6.3) | | 4 (0.4) | |  | |  | |
| Lung transplant | 5 (0.4) | | 5 (1.2) | | 0 (0) | |  | |  | |
| Other procedure | 51 (3.8) | | 23 (5.6) | | 28 (3.0) | |  | |  | |
| Duration of CPB (minutes) | | 71 (53.25-100) | | 94 (66-131) | | 65 (50-86) | | < 0.001* | | 0 |
| Aortic cross-clamping time (minutes) | | 47 (34-67) | | 61 (39-86) | | 43 (32-59) | | < 0.001* | | 0 |
| Temperature nadir (°C) | | 36 (35-36) | | 35.80 (34.78-36) | | 36 (35.30-36) | | < 0.001* | | 8 |
| Procedure with circulatory arrest | | 40 (3.0) | | 20 (4.9) | | 20 (2.1) | | 0.007 | | 0 |

***NOTE.*** *Prior analysis, 132 samples with haemolysis were excluded. Values are expressed as numbers (%) or median (interquartile range).****Abbreviations:*** *HBP, heparin binding protein, CABG, coronary artery bypass graft; AVR, aortic valve replacement; CPB, cardiopulmonary bypass.*

***Statistical tests:*** *Chi-Square test and Mann Whitney U test (*).*

**Supplementary Table 5. *Sensitivity analysis - postoperative characteristics of the study population***

| **Variable** | **All**  (n = 1348) | **HBP > 41** (n = 411) | **HBP** ≤ **41** (n = 937) | ***p*** | **Missing** |
| --- | --- | --- | --- | --- | --- |
|  |  |  |  |  |  |
| Peak CKMB concentration (µg/L) | 17 (12-28) | 23 (15-44) | 16 (11-23) | < 0.001* | 5 |
| Peak lactate concentration (mmol/L) | 2.20 (1.70-2.90) | 2.50 (1.90-3.40) | 2.10 (1.60-2.70) | < 0.001* | 5 |
| Peak CRP concentration (mg/L) | 119 (55-190) | 139 (80-206) | 102 (47-182.75) | < 0.001* | 905 |
| Peak WBC count (x10^9^/L) | 12.6 (10.4-15.2) | 13.6 (10.9-17.2) | 12.25 (10.28-14.60) | < 0.001* | 184 |
| Peak creatinine concentration (µmol/L) | 97 (81-128) | 107 (87-168) | 94 (79.5-119) | < 0.001* | 5 |
| Total dose (μg) norepinephrine administered per kilogram. | 11.94 (2.68-46.67) | 29.66 (5.10-143.40) | 8.55 (2.01-31.12) | < 0.001* | 0 |
| Duration of norepinephrine administration (hours) | 15 (9-20) | 17 (11-31) | 14 (8-18) | < 0.001* | 0 |
| Norepinephrine administered > 24h | 245 (18.2) | 120 (29.2) | 125 (13.3) | < 0.001 | 0 |
| Total dose (mg) dobutamine administered per kilogram. | 0 (0-0.63) | 0 (0-2.31) | 0 (0-0.16) | < 0.001* | 0 |
| Duration of dobutamine administration (hours) | 0 (0-8) | 0 (0-16) | 0 (0-2.5) | < 0.001* | 0 |
| Dobutamine administered > 24h | 112 (8.3) | 65 (15.8) | 47 (5.0) | < 0.001 | 0 |
| Time on ventilator (hours) | 4.24 (2.95-6.86) | 5.12 (3.40-13.50) | 4.03 (2.80-5.96) | < 0.001* | 0 |
| > 48 hours on ventilator | 56 (4.2) | 39 (9.5) | 17 (1.8) | < 0.001 | 0 |
| Postoperative infection | 57 (4.2) | 32 (7.8) | 25 (2.7) | < 0.001 | 0 |
| Postoperative stroke | 31 (2.3) | 16 (3.9) | 15 (1.6) | 0.010 | 0 |
| Postoperative CRRT or dialysis | 41 (3.0) | 28 (6.8) | 13 (1.4) | < 0.001 | 0 |
| Postoperative multiple organ failure | 14 (1.0) | 11 (2.7) | 3 (0.3) | < 0.001 | 0 |
| Reoperation due to bleeding | 47 (3.5) | 19 (4.6) | 28 (3.0) | 0.132 | 0 |
| Postoperative IABP | 40 (3.0) | 20 (4.9) | 20 (2.1) | 0.007 | 0 |
| Postoperative atrial fibrillation | 473 (35.1) | 178 (43.3) | 295 (31.5) | < 0.001 | 0 |
| 30-days mortality | 15 (1.1) | 10 (2.4) | 5 (0.5) | 0.004 | 0 |

***NOTE.*** *Prior analysis, 132 samples with haemolysis were excluded. Values are expressed as numbers (%) or median (interquartile range).****Abbreviations:*** *HBP, heparin binding protein; CKMB, creatine kinase myocardial band; CRP, C-reactive protein; WBC, white blood cell; CRRT, continuous renal replacement therapy; IABP, intra-aortic balloon pump.*

***Statistical tests:*** *Chi-Square test and Mann Whitney U test (*).*
